# Proporties and Synthesis of Biosilver Nanofilms for Antimicrobial Food Packaging

**DOI:** 10.3390/polym15030689

**Published:** 2023-01-30

**Authors:** Gülay Baysal, Cihat Demirci, Haydar Özpinar

**Affiliations:** 1 Nutrition and Dietetics, Faculty of Health Sciences, Istanbul Aydin University, 34295 Istanbul, Turkey; 2 Food Engineering, Engineering Faculty, Istanbul Aydin University, 34295 Istanbul, Turkey

**Keywords:** antimicrobial food packaging silver nanofilms, organoclay, allicin and taurine extract, HPLC analysis, migration analysis

## Abstract

In this original research, biodegradable corn starch (CS) and wheat gluten (wg)-based silver nanofilms were synthesized and analyzed by using goji berry extract taurine (ta), garlic extract (GC), whey powder (wh), and montmorillonite clay nanoparticles. Antibacterial-corn-starch-based nano films were analyzed by using the methods of high-performance liquid chromatography (HPLC), Fourier Transform infrared spectroscopy (FTIR-ATR), X-ray diffraction (XRD), dynamic and mechanical (DMA) analysis, and scanning electron microscopy (SEM). In addition, the antibacterial resistances of the corn starch nano films against the bacteria *Salmonella* and *Staphylococcus aureus* (*S. aureus*) and *Listeria monocytogenes* were examined and the migration assays were carried out. The migration analysis results of CS_1_, CS_2_, and CS_3_ nanocomposite films were found as 0.305, 0.297, and 0.297 mg/dm^2^, respectively. The inhibition zone of CS_1_, CS_2_, and CS_3_ nanocomposite films were found as 1547, 386, and 1884 mm^2^ against *Salmonella* bacteria. The results show that silver nanofilms are suitable as packaging films for the production of packaging in milk and dairy products, liquid foods, and acidic foods.

## 1. Introduction

Goji berries have strong antioxidant features and a high level of beta carotene, iron, vitamins (C, B1, B6, and B2), and protein, as well as 18 amino acids and 21 minerals; moreover, they have more vitamin C than an oranger. They provide a feeling of satiety with its high fiber content of 21%. They are beneficial for treating high cholesterol and cancer since they protect retina cells and bones, etc., and they have bioactive benefits. It is also known that they provide energy, facilitate digestion, and are good for depression and anxiety disorders [1,2]. In this study, taurine was extracted from goji berries.

The taurine is a thiol-containing free amino acid that does not participate in the structure of proteins. As an essential amino acid and pharmacologically speaking, its clinical uses are expanding. The taurine extract, which is colorless, water-soluble, and strongly acidic due to its sulfonate group, has high antioxidant and antibacterial properties, much like whey powder [3,4,5].

As is known, the synthetic polymers used in food packaging contain components that are very harmful to the environment and human health. Petroleum-based synthetic polymers and derivatives cause serious damage to the environment due to the waste they transform. Plastic packaging materials, which are widely used in food packaging throughout the world, are a serious problem [6,7]. The mixing time of plastics with nature can last for centuries. In this sense, the need for biodegradable biopolymers is increasing day by day, especially in food packaging. However, their use in food packaging is limited due to the poor mechanical and barrier properties of biopolymers. Therefore, the biodegradable biopolymers are in great need of filling materials that improve their mechanical and barrier properties, as well as the bioactive components that enhance their antibacterial properties. Obtaining the preferred bioactive components from plant extracts, on the other hand, prevents the risks that may occur due to harmful migrations in food packages [8,9]. Additionally, antibacterial biopolymer packaging films, which can degrade in a short period of time without harming nature, are gaining importance [9,10]. In particular, antimicrobial food-packaging films prolong the shelf life of foods by inactivating the growth of pathogenic microorganisms in food products [11,12,13,14].

In this study, biopolymer food-packaging films, which display antimicrobial activity and contribute to the protection of nature in a short time, were synthesized. Initially, montmorillonite (Mt) clays were modified with taurine (ta) extracts obtained from goji berries dried at different temperatures, and the organoclays were synthesized. The resulting organoclays interacted with antibacterial garlic extract and whey powder, and corn starch and wheat gluten biopolymers. The antibacterial biopolymer packaging silver nanofilms, which have high antibacterial resistance, were synthesized. Synthesized silver nanofilms were analyzed by HPLC, FTIR, SEM, and DMA. The well diffusion method was applied to synthesized biofilms for antibacterial analyses for *S. aureus*, *Listeria monocytogenes*, and *Salmonella* bacteria, and migration analyses were performed. 

## 2. Materials and Methods

### 2.1. Materials

Garlic, silver nitrate (AgNO_3_), DPPH (1,1-diphenyl-2-picrylhydrazyl; CAS-No: 1898-66-4), methanol anhydrous (99.8%), nutrient agar, hydrochloric acid (37%), sodium borohydride, and sodium hydroxide were purchased from Sigma-Aldrich (St. Louis, MO, USA). Corn starch, goji berry fruit, wheat gluten, and whey powder were supplied by a local producer (Food Market in Istanbul/Turkey). Na^+^-montmorillonite was provided by Southern Clay Products Inc. (Gonzales, TX, USA). The physical and chemical properties of sodium montmorillonite had the following the chemical formulas: (Na, Ca)_0.33_ (Al, Mg)_2_ Si_4_O_10_ (OH)_2_ 6H_2_O, density: kg/dm^3^ 2.860, specific surface area: 0.750 m^2^/kg, and CEC: 920 meq/kg; their composition was wt. %: 1.40 Na, 2.44 Ca, 9.99 Al, 8.88 Mg, 20.7 Si, 35.53 O, and 0.37 H.

### 2.2. Preparation of Samples

#### 2.2.1. Preparation of Goji Berry Extract (Taurine)

The goji berry fruit was prepared by drying at 2 different temperatures—40 °C and 60 °C—for 24 h. The dried samples were ground into powder in a blender. The synthesis of the extracts of the obtained samples was done according to the literature [15]. The taurine extract obtained was analyzed by high-performance liquid chromatography (HPLC) analysis. As the final product, taurine extracts with different drying conditions, and ta40 and ta60 compounds as extracts, were obtained.

#### 2.2.2. The Synthesis of Organoclays

In order for the montmorillonite clay to gain an organic character, it was modified with taurine extract obtained from goji berries. The modification procedure was performed according to the literature [8]. The organoclays modified were named Mt-ta40 and Mt-ta60, respectively.

#### 2.2.3. The Synthesis of Silver Nanofilms

The synthesizing procedure of the silver nanofilms was carried out according to the literature [16]. The silver nanofilms were spread on glass slides (7.0 × 1.5 cm) and dried at room temperature for 10 days. The synthesis details of the silver nanofilms are shown in Table 1. The silver nanofilms were named CS_1_, CS_2_, and CS_3_, respectively.

#### 2.2.4. Characterization

The taurine extracts were analyzed by HPLC-DAD-UV-Vis. Acetonitrile and 0. 1% trichloroacetic acid were used as mobile phase. The PDA detector wavelength was 470 nm, and the emission wavelength was 530 nm. The flow rate was 1.0 mL/min, the injection volume was 20 µL, and the temperature was 35 °C. The surface morphologies of nanocomposites were examined by using a JOEL JSM 5600 LV scanning electron microscope (SEM) (Tokyo, Japan) with an accelerating beam at a voltage of 40 kV. The organic groups on the silver nanofilms were determined by FTIR-ATR. The mechanical properties of the cornstarch silver nanofilms were analyzed by using TA Instruments’ dynamic mechanical analyzer (DMA), Model 2980, New Castle, DE, USA).

#### 2.2.5. The Antibacterial Analysis

The bacteria *Listeria monocytogenes*, *S. aureus*, and *Salmonella* were used for the antibacterial analysis. The analysis procedure was applied according to the literature [17]. The nutrient agar was used as the medium.

#### 2.2.6. The Migration Analysis

##### The Contact Analysis

Migration analyses were performed by preparing 50% ethanol, 10% ethanol, and 3% acetic acid solutions.

- 50% ethanol; simulant imitating milk and milk products,
- 10% ethanol; simulant imitating liquid foods,
- 3% acetic acid; simulant imitating acidic foods.

The measurement method of the migration analysis has been carried out according to the literature [18], and TGK 2013/34 Article 8. The migration assays were carried out by using the procedures given in the Committee European Normalization Environment (CEN ENV) 1186 Prestandard.8 and TGK 2013/34 Article 8. 

#### 2.2.7. The Statistical analysis

For statistical analysis, the Minitab 16 software was used. ANOVA and Tukey’s test at a confidence level of 95% were used [12].

## 3. Results and Discussion

### 3.1. HPLC Analysis

**Figure 1:**
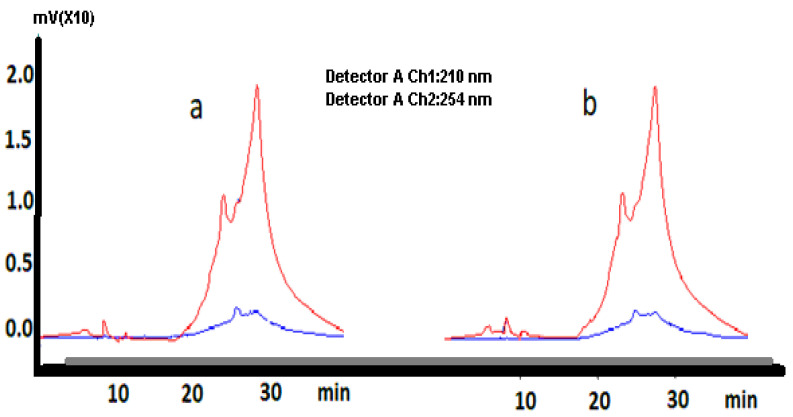
The HPLC analysis spectrum of goji berry extracts ta40 (**a**) and ta60 (**b**).

Figure 1 shows the HPLC analysis spectrum of goji berry extracts ta40 and ta60. The peaks seen in the HPLC spectrum around 5.0–5.5 min and 8 min prove the presence of taurine in goji berry extract [15].

### 3.2. FTIR-ATR Analysis

The FTIR-ATR spectra of orgaoclays showed peaks at 1668 cm^−1^ assigned to carbon–carbon and carbon–nitrogen stretching vibrations in taurine extract. The band of O-H represents water adsorption on the montmorillonite at 3627 cm^−1^. The stretching region of C-H is related to the modified organoclay molecules in the region of 2850–2928 cm^−1^. These bands are based on the C-H antisymmetric and symmetric stretching bonds. The amin peaks shown in the spectrum of the wheat gluten amide I have been defined as mainly arising from amide carbonyl stretching, a combination of amide NH bending and CH stretching that can be used to characterize the protein secondary structures. A peak at 1650 cm^−1^ is associated with α-helical and a random structure, and the shoulder at ≈1668 cm^−1^ is associated with β-turns and could also be related to glutamine side chains. The amide I region of the hydrated gluten protein broad shoulders was observed at 1654–1650 cm^−1^, indicating α-helix conformation [19,20,21,22,23,24].

Moreover, the peaks at wave numbers around 1300 and 1500 cm^−1^ may be interpreted to the stretching vibrations of C=C and C=O, respectively, which exist in the FTIR-ATR spectra of taurine and garlic-loaded polymer [8,11,12,13,14,15,16]. The fact that the peaks at 2928–2941–2820–2863 cm^−1^ are higher than the spectrum of pure starch proves that the modification made by taurine and garlic extracts was successful. Moreover, it can be assumed that the interactions resulting from the modification originated from aliphatic C-H tensile bands [15,17,18,19,20,21,22].

**Figure 2:**
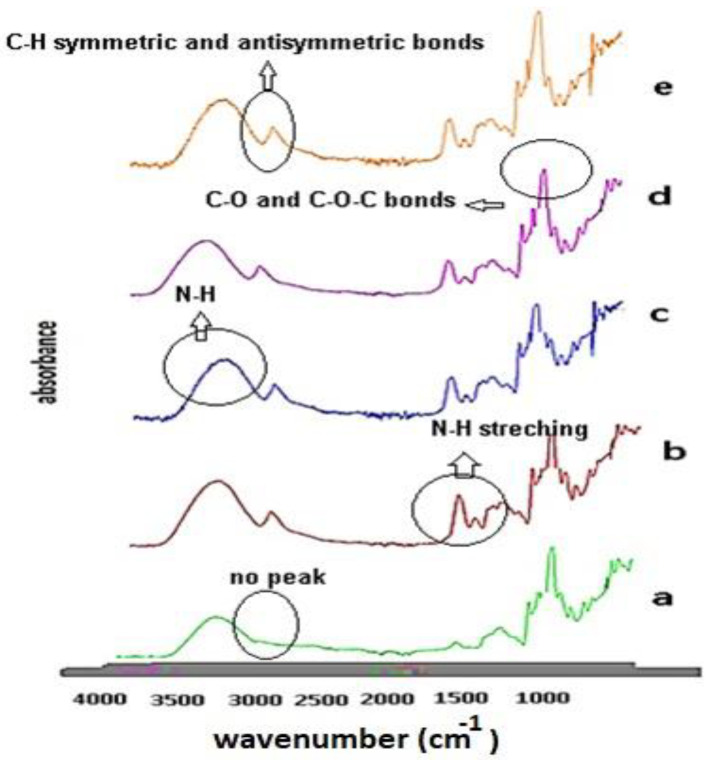
FTIR_ATR spectra of the corn starch (**a**),wheat gluten (**b**), CS_1_ (**c**), CS_2_ (**d**), and ve CS_3_ (**e**) silver nanofilms.

In the spectrum of the wheat gluten (Figure 2b), the peaks at the wavelengths of 1178–1021 cm^−1^ showed C-O stretching vibrations in the C-O-C groups. The broad band that appeared at 3272 cm^−1^ represented hydroxyl groups bonded with hydrogen and N-H stretching bands. The FTIR-ATR spectra of the silver nanofilms are shown in Figure 2c–e. The peaks at 1641–1643–1640 cm^−1^ in Figure 2 originated from interactions of the taurine and garlic extracts that were used to modify the clay. The peak at 1641 cm^−1^ also represented the C-O stretching that overlapped with the N-H stretching in the same region (amide II).

### 3.3. The Antibacterial Analysis

**Figure 3:**
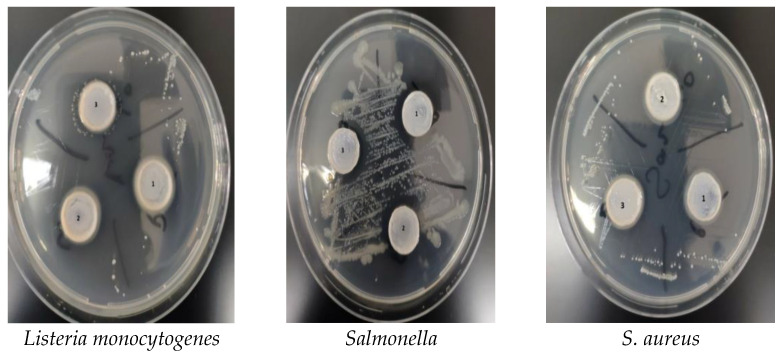
The inhibition zones of CS_1_ (1), CS_2_ (2)_,_ and CS_3_ (3) silver nanofilms against to *Listeria monocytogenes*, *Salmonella* and *S. aureus*, respectively.

Figure 3 and Table 2 show the inhibition zone analysis results for the silver nanofilms against the bacteria *Listeria Monocytogenes*, *Salmonella*, and *S. aureus*. As is known, the high antimicrobial resistance of nano films to prolonging the shelf life of foods is an effective parameter in packaging applications. CS_1_ and CS_3_ silver nanofilms showed maximum resistance to Salmonella, with an area of 1547.5 and 1884.3 mm^2^, respectively. In addition, it may assumed that the taurine and garlic extract showed higher antibacterial resistance, especially against *Salmonella* bacteria. According to the results of the antibacterial analysis, the whey powder and goji berry extract taurine dried at 60 °C created larger inhibition zones, whereas the goji berry extract taurine dried at 40 °C exhibited low antibacterial performance [25].

**Figure 4:**
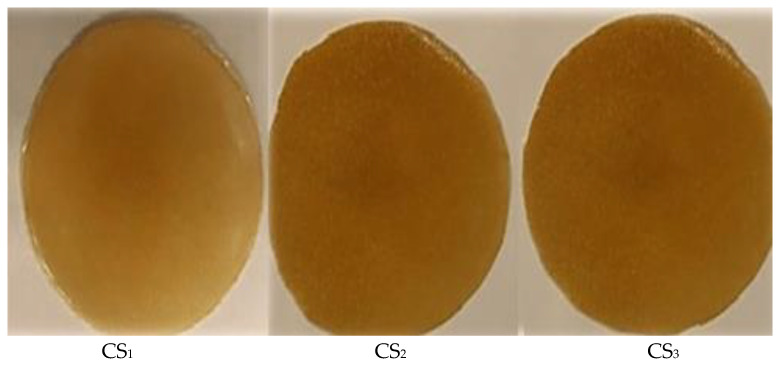
Images of obtained silver nanofilms.

*Salmonella* bacteria are found in non-typhoidal poultry, eggs, raw meat, and products made from raw eggs. Moreover, the milk and dairy products, egg products (pasteurized and frozen eggs, egg powder, etc.), meat and meat products, fishery products, live bivalve mollusks, live sea urchins, live tunicates, broth tablets and powders, soups in dry form, seasonings, cream, and other foods in powder and tablet form such as whipped cream and sauces can be given as examples of food types with a high risk of carrying *Salmonella* bacteria. For this reason, packaging syntheses that are resistant to *Salmonella* bacteria, which have such a high risk factor, are of great importance. Figure 4 shows images of obtained silver nanofilms.

### 3.4. SEM Analysis

**Figure 5:**
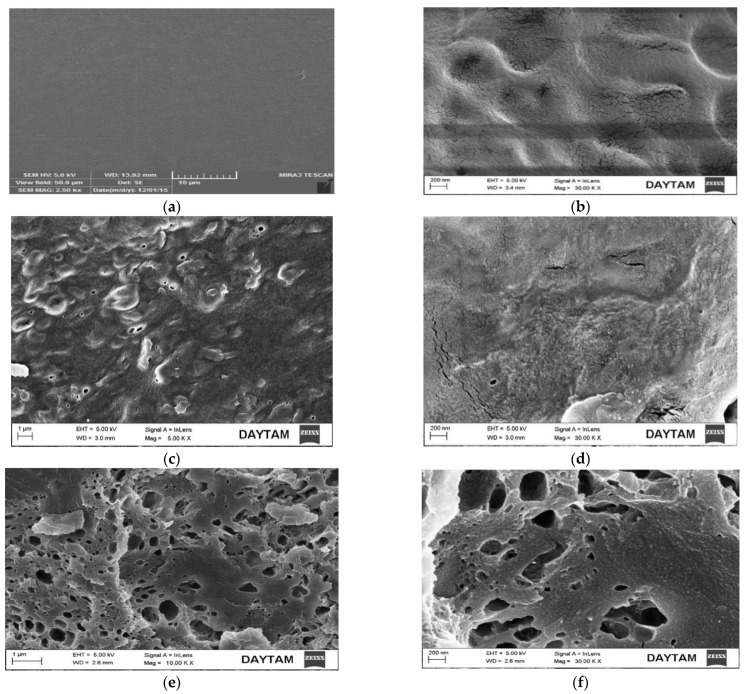

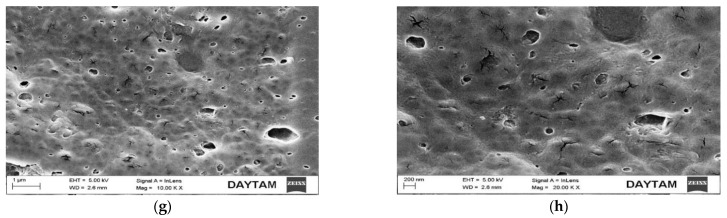
SEM images of corn starch CS (**a**), wheat gluten (**b**), CS_1_ (**c**,**d**), CS_2_ (**e**,**f**), and CS_3_ (**g**,**h**) nanocomposite films.

Figure 5 shows the surface morphology of the compounds. The silver nanofilms was measured through a JOEL JSM 5600 LV scanning electron microscope (SEM) with an accelerating voltage of 20 kV and a distance of 6 mm. The sample was fixed on the Ti/Al stub by double tape and coated with gold/palladium by a sputter coater for 90 s at 15 mA and 22 mbar pressure. In this study, solvents were evaporated from the environment during the boiling process in the nanofilm synthesis and plasticization stages. Therefore, the risks of keeping solvents in food packaging in electron spinning processes for SEM analysis were minimized. Thus, the migration of solvents to foods was prevented. The surface morphologies of silver nanofilms were observed between 200 nm and 1 µm. As is shown in Figure 5a,b, the surface morphology of the corn starch (CS) and wheat gluten were fluently smooth and homogeneous [26,27]. Figure 5 shows that the fractures formed as a result of the reactions occurring in the surface images of CS_1_ (c and d), CS_2_ (e and f), and CS_3_ (g and h) silver nanofilms, which formed a multilayered structure. These multi-layered structures also resulted in a rough morphology. The surface morphology of the samples was affected by the presence of taurine and garlic extract [26]. The addition of the nanoparticles resulted in a layered structure on the biomatrix surface, resulting in a stronger mechanical strength. The addition of extracts leads to an increase in tension and pressure on the polymer matrix surface, promoting the formation of strong bonds between the fibers [27]. The strong bonds formed also lead to an increase in the mechanical strength of the silver nanofilms. As is known, organoclays cause the formation of a layered structure on the polymer matrix surface. These results are also proof that the modification has taken place.

### 3.5. The Migration Analysis

**Figure 6:**
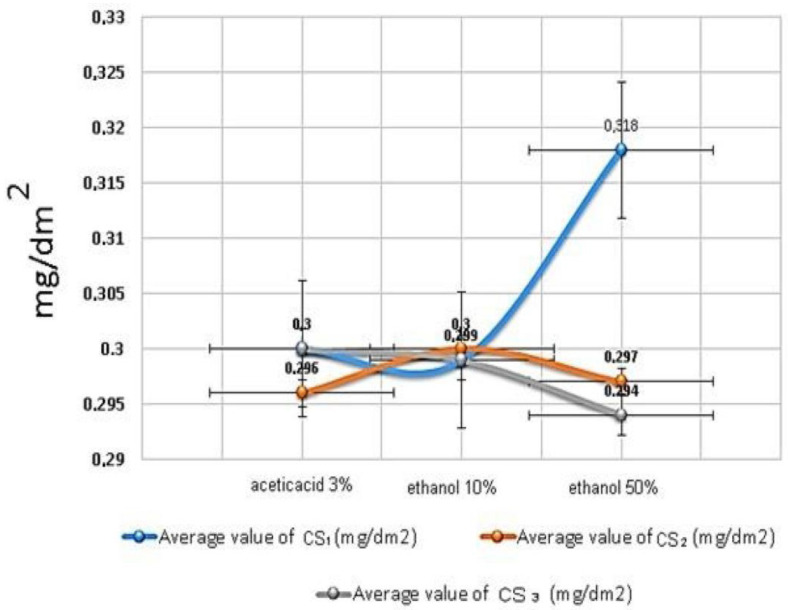
The migration analysis results of CS and CS silver nanofilms.

Table 3 and Figure 6 show the results of the migration and statistical analysis. According to the results, silver nanofilms can be used as food packaging material according to Article 8 of TGK 2013/34, and the analysis values are well below the reference values. Although the migration concentration of the materials is 6 dm^2^ of packaging material per 1 kg in accordance with the European Regulation 10/2011 (EC, 2011), according to TGK 2013/34 Article 8 in Turkey, the migration limit concentration of materials for foods is 10 mg/dm^2^ [18,28,29,30]. Figure 6 shows the migration analysis results of the CS silver nanofilms. As a result, it was proven by the analysis results that the CS_1_, CS_2_, and CS_3_ nanocomposite films were suitable to be packaging films for packaging production in milk and milk products, liquid foods, and acidic foods. It can be seen that all of the migration analysis results are quite below the standards values. If they are evaluated only according to the migration analysis results, it is possible to say that the synthesized silver nanofilms can be used in all food packaging, but the antibacterial analysis results limit this area, so it is recommend that they be used primarily in the food packaging of milk and dairy products.

This means that the CS silver nanofilms were found to be packaging films appropriate for usage with all food products. The concentration values of the silver nanofilm’s migration analysis were calculated according to the method described in a previously published article [18]. Table 4 shows the migration analysis results according to the literature.

### 3.6. The Dynamic and Mechanical Analysis

**Figure 7:**
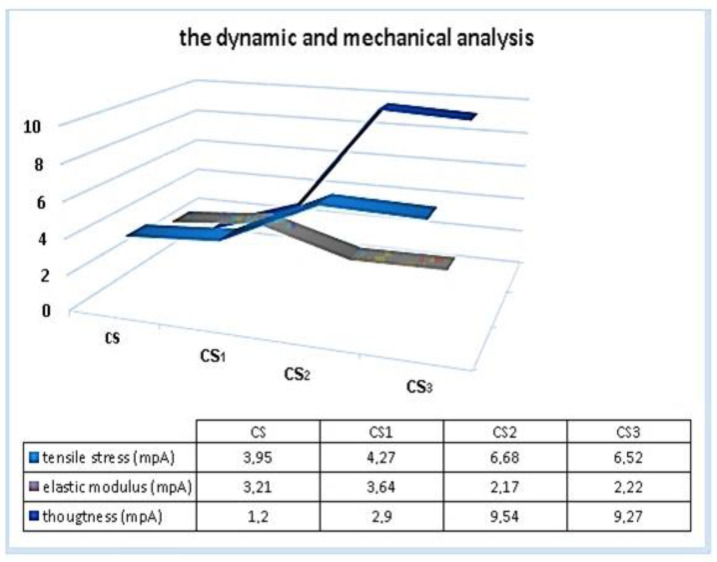
The tensile stress, elastic modulus and toughness analysis results of CS and CS silver naofilms.

The tensile stress (a), elastic modulus (b), and toughness (c) analysis results of the CS and CS silver nanofilms are shown in Figure 7. The tensile stress and the toughness of the CS silver nanofilms were enhanced compared to CS with no filler. The mechanical properties of corn starch silver nanofilms in the presence of montmorillonite clay, whey powder, and vegetable extracts improved. 

Figure 7 shows the tensile stress, elastic modulus, and toughness of pure CS, CS_1_, CS_2_, and CS_3_ silver nanofilms. According to the analysis results obtained, the tensile stress and toughness values of the CS_2_ and CS_3_ silver nanofilms showed more improvement than the CS_1_ silver nanofilm. The tensile stresses of CS_2_ and CS_3_ were increased by 68%, and the tensile stress of CS_1_ was increased by 8%, respectively. The reason for this can be said to originate from organoclay contained in the CS_2_ and CS_3_ silver nanofilms. As is known, organoclays are filling materials that support mechanical durability. In addition, even if the absence of organoclay in CS_1_ has lower tensile stress than CS_2_ and CS_3_, it can be said that it develops compared to the mechanical properties of pure CS biopolymer [17]. Moreover, the elastic modulus of CS_1_ showed more improvement than CS_2_, CS_3_, and pure CS. The increase in elastic modulus tends to decrease by 68% for the CS_2_ and CS_3_ silver nanofilms.

The presence of organoclay in the structure of the CS_2_ and CS_3_ silver nanofilms is a factor that reduces elasticity. The improvement of the mechanical properties of the synthesized silver nanofilms is also confirmed by scanning electron microscope images. The roughness of the surfaces is proof that their mechanical properties have improved. Figure 7 shows images of obtained silver nanofilms. The different concentrations of silver nitrate and sodium borohydride (0.3, 0.5, and 0.8 mM) caused darker color tones of the silver nanofilms.

Foods are exposed to contact materials including cutlery and dishes, containers, processing machine, and packaging materials during all steps passed from farm to fork. Food industry has been conducting research and development activities on food packaging to increase shelf life, keep the food quality at optimum level, attract consumer interests, and reduce waste. A package material for any type of food should minimize aroma and flavor losses, constitute an excellent barrier for gas and water, provide a perfect hermetically sealed seam, as well as have a good mechanical properties. Food contact materials including food packaging are generally based on paper, metal, ceramic, aluminum, lacquers and coating, and plastic.

Food packaging is used to increase shelf life, to keep food quality at optimum level, to attract consumer interest, to facilitate the sale and distribution. Foods packaging provides information to consumers on product name, brand name, net weight, manufacturer information, price, production date, as well as the nutrient values in addition to keeping food at the desired amount in a single vessel and making it easier to bring a number of units to be moved into a single cluster and use. Therefore, food industry makes expenditures on the research and development activities of food packaging systems. The degree of the final product quality and safety, and consumer expectations from the ergonomic features of the package affects the acceptance criteria of a package material. A package material for any type of foods should minimize aroma and flavor losses, constitute an excellent barrier for gas and water, provide a perfect hermetically sealed seam, as well as have a good mechanical properties and offer chemical and biological protection against contamination [31,32].

## 4. Conclusions

In this study, three different nanocomposite films—CS_1_, CS_2_, and CS_3_—were synthesized. As a result of the analyzes made, it was confirmed by the FTIR, SEM, HPLC, and DMA results that the modification produced successful results. According to the results of antimicrobial studies, it has been proven that garlic-plant extract provides positive resistance against bacteria. In the migration analysis results, it was confirmed that the synthesized antimicrobial biopolymer food-packaging films were made in accordance with Article 8 of TGK 2013/34. It has been determined that the pore structure of the CS_1_ film has less gas permeability than CS_2_ and CS_3_, thus creating a stronger gas barrier. This is because of the whey powder used in CS_1_ synthesis. The synthesized films will play an effective role in increasing the shelf life of the food products to be used. As a result, the migration analyses of the synthesized nanocomposite antimicrobial films show that they are suitable for use in food packaging. The successful results have shown that the synthesized films are not dangerous. In addition, it was concluded that the need for additives and chemical preservatives in foods can be reduced. In this way, the shelf life of foods is extended with natural bioactive ingredients.

## Figures and Tables

**Table 1:** The synthesis steps of nanocomposite films.

Samples (g)	CS_1_	CS_2_	CS_3_
Mta-40	-	1.0	-
Mtb-60	-	-	1.0
Taurine	1.0	1.0	1.0
Allicin	1.0	1.0	1.0
Wheat gluten	2.0	2.0	2.0
Whey powder	1.0	-	-
Corn starch	8.0	8.0	8.0

**Table 2:** The inhibition zones of silver nanofilms (mm^2^).

	*Listeria monocytogenes*	*Salmonella*	*S. aureus*
CS_1_	72.95 ± 0.27	1547.5 ± 3.16	382.02 ± 1.23
CS_2_	379.9 ± 1.39	386.88 ± 0.79	331.18 ± 1.07
CS_3_	35.87 ± 0.13	1884.3 ± 3.65	144.77 ± 0.46

Data are mean of triplicate measurements ± SD.

**Table 3:** The results of migration analysis.

Simulant	Acetic Acid3%	Ethanol 10%	Ethanol 50%
Chemical consumption (L)	0.15	0.15	0.15
Sample area (cm^2^)	70	70	70
Conditions (°C/h)	40 °C/10 day	40 °C/10 day	40 °C/10 day
Average value of CS_1_ (mg/dm^2^)	0.300 ± 0.0056	0.299 ± 0.0038	0.318 ± 0.0073
Average value of CS_2_ (mg/dm^2^)	0.296 ± 0.0042	0.300 ± 0.0033	0.297 ± 0.0056
Average value of CS_3_ (mg/dm^2^)	0.300 ± 0.0044	0.299 ± 0.0041	0.294 ± 0.0047

Data are mean of triplicate measurements ± SD.

**Table 4:** According to the literature of migration analysis results.

Simulant	Migration Ratio (mg/dm^2^)	References
Mt-GC-CS	3.65 ± 0.46	[6]
Plastic food containers	7.90	[18]
PET/PE	5.29 ± 0.67	[28]
PET/Alu/OPA/CPP	3.42 ± 0.10	[28]
nanosilver into food simulants	1.65–2.37	[28]
nanosilver into food simulants (A_2_–A_3_)	0.000032–0.000034	[10,11,12,13,14,15,16,17,18]
CS_1_, CS_2_, and CS_3_	0.300 ± 0.0033	(in this study)

## Data Availability

Not applicable.
